# Respiratory Viral Positivity Is Associated with Decreased Risk of Serious Bacterial Infections in Febrile Infants

**DOI:** 10.5811/westjem.53209

**Published:** 2026-09-24

**Authors:** Diormi A. Rosario, Anum Mitha, Jessica Zerzan, Sharmila Babu, Saxony Busta, Michael Silver, Antonios Likourezos, Christina Bravi, Hector Vazquez

**Affiliations:** * Maimonides Medical Center, Department of Emergency Medicine, Brooklyn, New York; † Children’s Hospital of Orange County, Department of Pediatrics, Orange, California; ‡ New York University Langone, Department of Pediatrics, New York, New York; § Duke University Hospital, Department of Pediatrics, Durham, North Carolina

## Abstract

**Introduction:**

Febrile infants ≤ 60 days of age are at increased risk for serious bacterial infections (SBIs), often necessitating extensive diagnostic evaluation and hospitalization. Although respiratory viral infections are common in this population and may influence the likelihood of concurrent bacterial infection, the relationship between respiratory viral panel (RVP) results and SBI risk remains an important consideration in clinical decision-making. The objective of this study was to evaluate the association between RVP results and the occurrence of SBIs among febrile infants ≤ 60 days of age.

**Methods:**

In this single-center, retrospective study we analyzed data from infants ≤ 60 days of age who presented to the emergency department with fever (temperature > 100.4 °F/38 °C) between September 2020–May 2024. Infants were divided into two subgroups based on RVP results: positive or negative for any viral infections via a multiplex polymerase chain reaction panel. The overall rate of SBI, and the rates of individual SBIs (urinary tract infection [UTI], bacteremia, and bacterial meningitis), were compared between groups. The primary outcome was the rate of SBIs among infants with any positive RVP compared to those with a negative RVP.

**Results:**

We analyzed 1,986 charts of febrile infants ≤ 60 days of age, of whom 485 (24.4%) met criteria. Of these, 309 (63.7%) had a positive RVP for at least one pathogen while 176 (36.3%) were all negative. Overall, the rate of serious bacterial infection in the RVP negative group was 35.8% (95% CI, 29%–43.1%) compared to 6.5% (95% CI, 4.1%–9.6%) in the RVP positive group (*P* < .001). Infants with a positive RVP had a significantly lower prevalence of serious bacterial infection (7.4%) and UTI (5.5%) compared to those with a negative RVP (*P* < .001). In the RVP positive group, 17 patients (5.5% [95% CI, 3.4%–8.5%]) had a UTI compared to 46 (26.1% [95% CI, 20.1%–33%]) in the RVP negative group. Notably, there were four cases of bacteremia (1.3% [95% CI, 0.4%–3%]) in the positive RVP group compared to 20 (11.4% [95% CI, 7.3%–16.7%]) in the negative RVP group. In the positive RVP group, there were 0 cases of meningitis (0% [95% CI, 0%–0.9%]) compared to 5 (2.8% [95% CI, 3.4%–85%]) in the RVP-negative group. The differences in bacteremia and meningitis rates between the two groups were statistically significant (*P* < .001).

**Conclusion:**

Febrile infants ≤ 60 days of age with a positive RVP are associated with a significantly lower risk for SBI compared to those with a negative RVP, a 64% relative risk reduction. If validated with a prospective study, these findings could help guide clinical decision-making, potentially allowing for a more nuanced approach to the management and disposition of febrile infants, particularly the need for invasive workup and hospitalization.

## INTRODUCTION

The evaluation of febrile infants, particularly those under two months of age, remains a subject of ongoing controversy in pediatric emergency medicine. Febrile illness is among the most common reasons for emergency department visits in infants < 60 days of age. Fever raises clinical concern for serious bacterial infections such as meningitis, bacteremia, or urinary tract infections (UTI).^6^ These infections can result in significant morbidity if not promptly diagnosed and treated.

Distinguishing between viral and bacterial etiologies in febrile infants in inherently challenging due to overlapping clinical presentations. To address this, the American Academy of Pediatrics (AAP) developed widely adopted guidelines for the evaluation and management of febrile infants.^9^ These guidelines have played a crucial role in improving clinical outcomes, thereby reducing the use of unnecessary invasive procedures in this vulnerable population. However, a notable limitation of these guidelines is the omission of respiratory viral panel (RVP) testing. The RVP is a diagnostic tool that can identify a wide range of viral pathogens, including influenza, parainfluenza, coronavirus, adenovirus, human metapneumovirus, human rhinovirus/enterovirus, and respiratory syncytial virus.^2^ Viral etiologies may decrease the likelihood of a concurrent serious bacterial infection; consequently, identifying them may reduce reliance on invasive diagnostic testing and empiric broad-spectrum antibiotics. Incorporating RVP testing into the evaluation of febrile infants has the potential to enhance diagnostic precision, improve patient outcomes, and optimize the use of healthcare resources.

## METHODS

This retrospective cohort study included febrile infants ≤ 60 days of age who presented to the pediatric emergency department (PED) from September 2020–May 2024. We used stratified sampling to derive data.^15^ Fever was defined as any documented temperature ≥ 100.4 ^o^ F in the PED, a prior healthcare setting, or at home within the preceding 24 hours. We identified eligible patients through the electronic health record (EHR) including all infants ≤ 60 days of age with a RVP collected. The exclusion criteria were as follows: preterm infants (< 37 weeks’ gestation); infants < 14 days of age whose perinatal course was complicated by maternal fever, infection, and/or antimicrobial use; febrile infants with high suspicion of herpes simplex virus infection (eg, infants with vesicles); infants with a focal bacterial infection (eg, cellulitis, omphalitis, septic arthritis, osteomyelitis); infants with documented or suspected immunodeficiency; infants whose neonatal course was complicated by surgery or infection; infants with congenital or chromosomal abnormalities; medically fragile infants who required some form of technology or ongoing therapeutic intervention to sustain life; and infants who had received immunizations within the prior 48 hours. Additionally, patients were excluded if they had not undergone both RVP testing and culture collection (urine, blood, and/or cerebral spinal fluid).

**Population Health Research Capsule**

What do we already know about this issue?

*Febrile infants ≤ 60 days are at risk for serious bacterial infections, but the role of respiratory viral testing in risk stratification remains unclear*.

What was the research question?

*Does a positive respiratory viral panel reduce serious bacterial infection risk in febrile infants ≤ 60 days old?*

What was the major finding of the study?

*A positive respiratory viral panel was associated with a 64% lower serious bacterial infection risk (RR 0.34)*.

How does this improve population health?

*Respiratory viral testing testing may improve risk stratification, reducing unnecessary invasive testing, antibiotics, and hospitalizations in this population*.

Variables collected included demographics, chief complaint, presenting symptoms, maximum temperature, ED disposition, diagnostic codes, antibiotic use, and hospital length of stay (if admitted). Laboratory data included white blood cell count, absolute neutrophil count, C-reactive protein, procalcitonin, urinalysis, urine culture, blood culture, cerebrospinal fluid culture, and RVP results. The RVP test used was the BioFire Respiratory 2.1 Panel (BioFire Diagnostics LLC, Salt Lake City, UT), which detects 21 respiratory pathogens (adenovirus, coronavirus HKU1, coronavirus NL63, coronavirus 229E, coronavirus OC43, human metapneumovirus, human rhinovirus, and enterovirus**)**. We collected the data from the EHR and stored it using Microsoft Excel (Microsoft Corp, Redmond, WA). The primary outcome was the presence of serious bacterial infection, defined as a UTI, bacteremia, or bacterial meningitis. A UTI was defined as a urine culture positive with at least 50,000 colonies/mL of a single organism obtained via sterile catheterization. Meningitis and bacteremia were defined as growth of a single pathogen on either cerebrospinal fluid or blood culture. Enteritis was not included as part of a serious bacterial infection as we do not routinely perform stool cultures at our institution.

We categorized patients based on RVP results (positive versus negative) and compared the overall rate of serious bacterial infections between the groups. Continuous variables were summarized with medians and interquartile ranges. Categorical variables were summarized with frequencies and percentages. When comparing categorical variables, we used the Fisher exact test and chi-square test. All statistical analysis was carried out with SPSS statistical software (IBM Corporation. Armonk, NY) with a significance threshold of 0.05. The study was approved by the institutional review board at Maimonides Medical Center.[Table t1-wjem-27-1317]

## RESULTS

A total of 485 (24.4%) patients met inclusion criteria after we analyzed the charts of 1,986 febrile infants who presented to the ED from September 2020–May 2024. All patients had at least one culture collected (urine, blood, and/or cerebrospinal fluid). All patients but 12 (2.5%) had an extended RVP collected. The rate of serious bacterial infection in the RVP negative group was 35.8 % (95% CI, 29%–43.1%) compared to 6.5 % (4.1%–9.6%) in the RVP positive group (*P* < .001). Of the serious bacterial infection in the RVP negative group, 46 patients had a UTI, 20 had bacteremia, and five had bacterial meningitis. A statistical significance was observed for serious bacterial infection in the RVP-positive group when compared to the RVP-negative group (Table 2). In the RV-positive group, four patients had bacteremia, and zero had meningitis. Of the patients with bacteremia, three were > 29 days and one < 28 days of age. The organisms involved in the cases of bacteremia include *Escherichia coli* (two), *Acinetobacter ursingii*, and *Streptococcus lutetiensis*. Of the four patients with bacteremia, the ones with *E coli* were likely true bacteremia; however, the patients with *A ursingii, and S lutetiensis* may have been secondary to contamination.

## DISCUSSION

The utility of RVP testing in the risk stratification of febrile infants ≤ 60 days old has been a subject of ongoing clinical debate. Given the vulnerability of this population to serious bacterial infections, the diagnostic evaluation frequently involves invasive tests and empiric antibiotics. The development of the AAP guidelines has resulted in improved risk stratification and contributed to a reduction of invasive and antibiotics for febrile infants. Prior studies have shown that patients with influenza, respiratory syncytial virus, or severe acute respiratory syndrome coronavirus 2 are at lower risk for serious bacterial infection. Our study builds upon this body of evidence by evaluating whether a positive RVP result is associated with a decreased likelihood of serious bacterial infections compared with infants who test RVP negative.

Our findings indicate that a positive RVP result is significantly associated with a lower risk for serious bacterial infection. Notably, no cases of bacterial meningitis were identified in the RVP-positive group, further supporting the potential protective nature of viral detection. Although the AAP guidelines have provided a structured approach to the evaluation of febrile infants, they do not currently incorporate RVP testing as a factor in clinical decision-making. The presents study supports the growing body of evidence suggesting that a positive RVP test may be a protective factor against serious bacterial infection in febrile infants and could help minimize invasive testing and of broad-spectrum antibiotics in this vulnerable population.

## LIMITATIONS

This study has several limitations. First, it was conducted at a single center, thereby limiting generalizability. Moreover, because not all hospitals have access to rapid RVP testing there is a limit to the utility of the test in real-time clinical decision-making. Secondly, the retrospective design is inherently subject to biases such as incomplete documentation and unmeasured cofounding factors. In addition, the sample size was small, making it less likely to discern differences in rare outcomes such as bacterial meningitis and bacteremia. These limitations underscore the need for larger, prospective multicenter studies to evaluate the role of RVP testing more precisely in the risk stratification of febrile infants, especially in the context of coexisting viral infections.

## CONCLUSION

Febrile infants ≤ 60 days old with a positive respiratory viral panel are associated with a significantly lower risk for serious bacterial infections compared to those with negative RVP results, corresponding to a 64% relative risk reduction (0.34). These findings support the potential utility of RVP testing in guiding clinical decision-making, potentially allowing for a more nuanced approach to the testing, management, and disposition of febrile infants.

## Figures and Tables

**Table 1 t1-wjem-27-1317:** Baseline characteristics of patients by respiratory viral panel (RVP) status in a study examining the rate of serious bacterial infections among febrile infants with a positive RVP.

	RVP Negative (n = 176)	RVP Positive (n = 309)	*P*-value
Age (days)	27 (24 – 29)	35 (33 – 37)	<.001
Sex			
Male	108 (38.6%)	172 (61.4%)	.26
Female	68 (33.2%)	137 (66.8%	.26
Race			
Asian	37 (56.1%)	29 (43.9%)	< .001
Black	3 (60%)	2 (40%)	.36
Hispanic	26 (46.4%)	30 (53.6%)	.13
White	86 (30.9%)	192 (69.1%)	.01
Other	19 (27.9%)	49 (72.1%)	.16
Maximum Temperature (ºF)	101.5 (101.4 – 101.7)	101.3 (101.2 – 101.4)	<.001
White blood cell count (x103/UL)	12.58 (11.44 – 13.72)	10.76 (10.26 – 11.26)	<.001

*RVP*, respiratory viral panel.

**Table 2:** Prevalence of serious bacterial infection, urinary tract infection, bacteremia, and meningitis by respiratory viral panel status, in a study of febrile infants.

		RVP negative	RVP positive	*P*-value
Total	485	176 (36%)	309 (64%)	<.001
SBI	83	63 (76%)	20 (24%)	<.001
UTI	63	46 (73%)	17 (27%)	<.001
Bacteremia	24	20 (83%)	4 (17%)	<.001
Meningitis	5	5 (100%)	0	.003

*RVP*, respiratory viral panel; *SBI*, serious bacterial infection; *UTI*, urinary tract infection.
